# Transactions of the Association of State Boards of Dental Examiners

**Published:** 1883-09

**Authors:** Geo. H. Cushing

**Affiliations:** Niagara Falls


### PROCEEDINGS.

### Transactions of the Association of State Boards of Dental Examiners.

NIAGARA FALLS, August 6, 1883.

Pursuant to adjournment, the Convention held at Lexington, Ky., Feb. 20, 1883, of various State Boards of Examiners, met at the Cataract House, at 2:30 P. M., of this day.

THE FOLLOWING STATE BOARDS WERE REPRESENTED:

Iowa, by Dr. W. P. Dickinson, of Dubuque.

Vermont, by Drs. James Lewis, of Burlington, and G. H. Swift, of Manchester.

Indiana, by Drs. P. G. C. Hunt, of Indianapolis, and S. B. Brown, of Fort Wayne, and M. H. Chappell, of Knightstown.

New Jersey, by Dr. J. Hayhurst, of Lambertsville.

Pennsylvania, by Dr. C. N. Peirce, of Philadelphia.

Ohio, by Dr. H. A. Smith, of Cincinnati, and Drs. Butler, of Cleveland, J. Taft, of Cincinnati, I. Williams, New Philadelphia, and F. H. Rehwinkel, of Chillicothe.

Michigan, by Drs. G. R. Thomas, of Detroit, and J. A. Robinson, of Jackson.

Illinois, by Drs. G. H. Cushing and A. W. Harlan, of Chicago.

Georgia, by J. N. Coyle, of Thomasville, and G. W. McElhaney, of Columbus.

On motion of Dr. Smith, a temporary organization was effected, and Dr. J. Taft was chosen Chairman, and Geo. H. Cushing, Secretary.

On motion of Dr. Peirce, it was voted, that each Board should cast ten votes, and that the ten votes should be equally divided between the members of any Board present.

On motion of Dr. Peirce, it was voted to proceed to effect a permanent organization.

On motion, the following were appointed a committee to draft a Constitution: Geo. H. Cushing, C. N. Peirce, and H. A. Smith.

A recess was taken to enable the committee to prepare their report.

The committee reported a draft of a Constitution which was voted upon, section by section, and finally adopted as a whole, as follows:

CONSTITUTION.

NAME.—This organization shall be known by the name of the National Association of Dental Examiners.

ART. II.

OBJECTS.—The objects of this Association, shall be to secure through the operation of the various State Examining Boards, a high and uniform standard of qualification for dental practitioners, and so far as practicable, uniformity of methods in the working of these Boards, and of legislation in creating them.

ART. III.

MEMBERS.—This Association shall consist of such different State Boards of Dental Examiners as may elect to join this National Association. They may be represented either by a delegate or delegates duly authorized, or by the whole Board. Certificates from the proper officers of any Board will be necessary to entitle such Board to representation in this body.

ART. IV.

VOTES.—Each State Board shall be entitled to ten votes. If at any meeting of this Association, but one member of any Board be present, he shall cast the whole number. In case there is more than one member present, the ten votes of that Board shall be equally distributed among and cast by those members of said Board who are present.

ART. V.

DUES.—Each State Board becoming connected with this Association shall pay annually to the Treasurer the sum of five dollars.

ART. VI.

OFFICERS.—The officers of this Association shall be a President, Vice-President and Secretary, and Treasurer. The last named two officers combined in one. They shall be elected by ballot, without nomination, and shall hold their appointments for one year, or until their successors are elected and qualified. A majority of all the votes cast shall be necessary to a choice.

ART. VII.

DUTIES OF OFFICERS.—The President shall preside at all the meetings according to parliamentary usage, as laid down in Cushing's Manual. The Vice-President shall perform the duties of the President, in case of the latter's absence or inability. The Secretary and Treasurer shall keep correct minutes of the proceed ings, give due notice of meetings, and attend to the necessary correspondence. He shall receive and hold all moneys belonging to the Association, and from them shall pay all drafts of the President, countersigned by the Secretary. His accounts shall be audited by a committee of three, appointed annually for that purpose.

ART. VIII.

OBLIGATIONS OF MEMBERS.--All State Boards belonging to this Association, shall be bound by its action, so long as they continue members of it.

Any Board refusing to be bound by the action of this body, shall from that time cease to be a member thereof.

ART. IX.

QUORUM.—The representatives of five State Boards shall constitute a quorum for the transaction of business.

ART. X.

MEETINGS.—There shall be held annually a meeting of this Association, at such time and place, as the Association may determine.

The President may call a meeting at any time during the year, upon the written request of five State Boards.

ART. XI.

AMENDMENTS.—Amendments to this Constitution may be made at any annual meeting, by the consent of all the members present. In case of any opposition, notification in writing shall be made of any such proposed change, and shall be laid over for one year, for final action, when the amendment can only be adopted by an affirmative vote of three-fourths of the voters present.

On motion, the election of officers was proceeded with, resulting in the choice of Dr. J. Taft, of Cincinnati, as President; Dr. G. W. McElhaney, of Columbus, Georgia, Vice-President; Dr. Geo. H. Cushing, of Chicago, Secretary and Treasurer.

On motion, adjourned, till 8 P. M.

The Association met pursuant to adjournment, President Taft in the chair.

The minutes of the afternoon session were read and approved.

Dr. Cushing moved that this Association recommend to the different State Boards, a standard for examination, which shall be twenty-five per cent higher than that adopted at Lexington.

Discussed by Drs. Hunt, Chappell, Thomas, Lewis, Hayhurst, Taft, Smith, Rehwinkel, Williams, McElhaney, Harlan and Peirce.

It was then adopted.

On motion of Dr. Peirce, the following Committee was appointed to present subjects for the consideration of the Association for to-morrow:

Drs. Peirce, McElhaney, and Lewis.

Adjourned till 8 o'clock A. M. to-morrow.

TUESDAY, Aug. 7, 8 A. M.

Association called to order by the President, Dr. Taft,

The minutes were read and approved.

The following members then paid their dues:

Pennsylvania, Ohio, Illinois, Vermont, Indiana, Iowa, Michigan, and Georgia.

The Committee appointed yesterday, to report subjects for consideration, this morning submitted their report in the form of the following resolutions:

*Resolved*, That a Committee of two, to consist of the President and Vice-President, be appointed, to act with the Secretary, in carrying out the requests of this Association.

*Resolved*, That this Association enjoin its members to accept the diplomas of no college, which does not require two full regular courses of lectures—or their equivalent—one full course and five years practice, as a pre-requisite for graduation.

*Resolved*, That this Association insists that no Board now connected with this body, shall confer degrees or titles of any nature.

*Resolved*, That this Association recommend to the State Societies, of all States, where no law exists, the necessity for an immediate effort to secure such legislation.

*Resolved*, That this Association furnish all State Societies with copies of a well digested law, so that uniformity in legislation, as far as practicable, may be attained.

Dr. Thomas offered the following:

*Resolved*, That this Association recommend to the various State Boards of Examiners, a form of certificate, to be issued by said State Boards to those, found upon examination, to be qualified to practice dentistry.

On motion of Dr. Peirce, it was voted, that where no objection was raised, the vote upon the foregoing resolutions should be taken *viva voce*, but if objection is made, then the vote should be taken upon the calling of the States

After some general discussion, the above six resolutions were adopted, in the order in which they appear, by a *viva voce* vote.

On motion of Dr. Harlan, the following Committee was appointed to draft a law for recommendation to the various States, desiring to procure such legislation.

Drs. Harlan, Taft, Peirce, Coyle, and Lewis.

On motion, adjourned, till 4:30 P. M.

TUESDAY, Aug. 7, 4:30 P. M.

The Association met pursuant to adjournment.

Dr. Taft in the chair.

The minutes were read and approved.

The bill of Dr. J. Taft, for printing proceedings of Lexington meeting amounting to seventeen dollars, was ordered paid.

The draft of a law, to be recommended to the different States, was then adopted, as follows:

AN ACT.

To insure the better education of practitioners of dental surgery, and to regulate the practice of dentistry in the State of ————.

SECTION I.

Be it enacted, by the people of the State of ————, represented in the General Assembly, that it shall be unlawful for any person who is not at the time of the passage of this Act, engaged in the practice of dentistry in this State, to commence such practice, unless he or she shall have obtained a certificate as hereinafter provided.

SEC. II.

A Board of Examiners to consist of five practicing dentists, is hereby created, whose duty it shall be to carry out the purposes and enforce the provisions of this Act.

The members of said Board shall be appointed by the Governor, who shall select them from ten candidates whose names shall be furnished him by the State Dental Society. Three members at least of this Board shall be members of the State Dental Society.

The term for which the members of said Roard shall hold their offices, shall be five years, *except* that the members of the Board first to be appointed under this Act, shall hold their offices for the term of one, two, three, four and five years respectively, and until their successors shall be duly appointed.

In case of a vacancy occuring in said Board, such vacancy shall be filled by the Governor from names presented to him by the ———— State Dental Society.

It shall be the Duty of the ——— State Dental Society, to present twice the number of names to the Governor, of those to be appointed.

Sec. III.

Said Board shall choose one of its members, President, and one the Secretary, thereof, and it shall meet at least once in each year, and as much oftener, and at such times and places, as it may deem necessary. A majority of said Board shall at all times constitute a quorum, and the proceedings thereof shall at all reasonable times be open to public inspection.

Sec. IV.

Within six months from the time that this act takes effect, it shall be the duty of every person, who is at that time engaged in the practice of dentistry, in this state, to cause his or her name and residence, or place of business, to be registered, with said Board of Examiners, who shall keep a book for that purpose.

The statement of every such person shall be verified under oath, before a Notary Public or Justice of the Peace, in such manner as may be prescribed by the Board of Examiners.

Every person who shall so register with said Board, as a practitioner of dentistry, may continue to practice the same as such, without incurring any of the liabilities, or penalties provided in this act, and shall pay to the Board of Examiners for such registration a fee of one dollar.

It shall be the duty of the Board of Examiners to forward to the County Clerk of each county, in the state, a certified list of the names of all persons residing in his county, who have registered in accordance with the provisions of this act; and it shall be the duty of all County Clerks to register such names in a book to be kept for that purpose.

Sec. V.

Any and all persons who shall so desire may appear before said Board, at any of its regular meetings and be examined with reference to their knowledge and skill in dental surgery; and if the examination of any such person or persons shall prove satisfactory, to said Board, the Board of Examiners shall issue to such persons, as they shall find to possess the requisite qualifications, a certificate to that effect in accordance with the provisions of this act. Said Board shall also endorse as satisfactory diplomas from any reputable dental college, when satisfied with the character of such institution, upon the holder of such diploma furnishing evidence satisfactory to the Board, of his or her right to the same.

All certificates issued by said Board shall be signed by its officers, and such certificates shall be prima facie, evidence of the right of the holder to practice dentistry in the State of——

Sec. VI.

Any person who shall violate any of the provisions of this act, shall be deemed guilty of a misdemeanor, and upon conviction may be fined not less than fifty dollars, or more than two hundred dollars, or be confined six months in the County Jail.

All fines received under this act shall be paid into the Common School Fund, of the county, in which such conviction takes place.

Sec. VII.

In order to provide the means for carrying out and maintaining the provisions of this act, the said Board of Examiners may charge each person, applying to, or appearing before them, for examination for a certificate, of qualification, a fee of ten dollars, which fee shall in no case be returned; and out of the funds coming into the possession of the Board, from the fees so charged, the members, of said Board, may receive as compensation, the sum of five dollars, for each day actually engaged in the duties of their office, and all legitimate and necessary expenses incurred in attending the meetings of said Board. Said expenses shall be paid from the fees and penalties received by the Board under the provisions of this act. And no part of the salary or other expenses of the Board shall ever be paid out of the State Treasury.

All moneys received in excess of said per diem allowance, and other expences, above provided for, shall be held by the Secretary of said Board, as a special fund, for meeting the expenses of said Board and carrying out the provisions of this act, he giving such bond as the Board shall from time to time direct.

And said Board shall make an annual report of its proceedings to the Govenor by the 15th of December of each year, together with an account of all moneys received and disbursed by them pursuant to this act.

### Sec. VIII.

Any person who shall receive a certificate of qualification from said Board shall cause his or her certificate to be registered with the County Clerk, of any county, or counties in which such persons may desire to engage in the practice of dentistry, and the County Clerks of the several counties in this state shall charge for registering such certificates, a fee of twenty-five cents for such registration.

Any failure, neglect or refusal on the part of any person holding such certificate to register the same with the County Clerk, as above directed, for a period six of months, shall work a forfeiture of the certificate, and no certificate when once forfeited shall be restored except upon the payment to the said Board, of Examiners, of the sum of twenty-five dollars as a penalty for such neglect, failure, or refusal.

### Sec. IX.

Any person who shall knowingly and falsely claim or pretend to have or hold a certificate of license, diploma, or degree granted by any society, or who shall falsely and with intent to deceive the public, claim or pretend to be a graduate from any incorporated Dental College, not being such graduate, shall be deemed guilty of a misdemeanor, and shall be liable to the same penalty as provided in section VI. of this act.

---

On motion the time and place of our next meeting was left to be fixed by the Officers of the Association.

On motion a cordial and earnest invitation was extended to all State Boards of Dental Examiners to join this Association and co-operate with us in our work.

On motion the Secretary was instructed to publish the proceedings of this Association and to forward copies to all State Boards of Examiners.

The following resolution was adopted:

*Resolved,* That all resolutions adopted by this Association at this meeting, are offered to the different State Boards of

Examiners as bearing especially upon the future actions of the Boards.

The minutes were then read and approved, and the Association adjourned.

GEO. H. CUSHING,
Secretary.

